# miR-214-Dependent Increase of PHLPP2 Levels Mediates the Impairment of Insulin-Stimulated Akt Activation in Mouse Aortic Endothelial Cells Exposed to Methylglyoxal

**DOI:** 10.3390/ijms19020522

**Published:** 2018-02-09

**Authors:** Cecilia Nigro, Paola Mirra, Immacolata Prevenzano, Alessia Leone, Francesca Fiory, Michele Longo, Serena Cabaro, Francesco Oriente, Francesco Beguinot, Claudia Miele

**Affiliations:** URT Genomics of Diabetes-IEOS, CNR & Department of Translational Medicine, Federico II University of Naples, Via Pansini 5, 80131 Naples, Italy; cecilia.nigro@alice.it (C.N.); paolamirra.lib@libero.it (P.M.); imma.prevenzano@libero.it (I.P.); aleleone86@libero.it (A.L.); francesca-976@libero.it (F.F.); mi_longo@libero.it (M.L.); serenacabaro@gmail.com (S.C.); foriente@unina.it (F.O.); beguino@unina.it (F.B.)

**Keywords:** miRNAs, endothelium, insulin resistance, diabetes mellitus, methylglyoxal

## Abstract

Evidence has been provided linking microRNAs (miRNAs) and diabetic complications, by the regulation of molecular pathways, including insulin-signaling, involved in the pathophysiology of vascular dysfunction. Methylglyoxal (MGO) accumulates in diabetes and is associated with cardiovascular complications. This study aims to analyze the contribution of miRNAs in the MGO-induced damaging effect on insulin responsiveness in mouse aortic endothelial cells (MAECs). miRNA modulation was performed by transfection of specific miRNA mimics and inhibitors in MAECs, treated or not with MGO. miRNA-target protein levels were evaluated by Western blot. PH domain leucine-rich repeat protein phosphatase 2 (PHLPP2) regulation by miR-214 was tested by luciferase assays and by the use of a target protector specific for miR-214 on PHLPP2-3′UTR. This study reveals a 4-fold increase of PHLPP2 in MGO-treated MAECs. PHLPP2 levels inversely correlate with miR-214 modulation. Moreover, miR-214 overexpression is able to reduce PHLPP2 levels in MGO-treated MAECs. Interestingly, a direct regulation of PHLPP2 is proved to be dependent by miR-214. Finally, the inhibition of miR-214 impairs the insulin-dependent Akt activation, while its overexpression rescues the insulin effect on Akt activation in MGO-treated MAECs. In conclusion, this study shows that PHLPP2 is a target of miR-214 in MAECs, and identifies miR-214 downregulation as a contributing factor to MGO-induced endothelial insulin-resistance.

## 1. Introduction

Type 2 diabetes mellitus (T2DM) is a chronic metabolic disease characterized by defects in both β-cell function and insulin sensitivity, which result in elevated blood glucose levels [1]. Diabetes-induced endothelial dysfunction is a critical and initiating factor in the genesis of diabetic vascular complications. Several mechanisms of endothelial dysfunction in T2DM have been identified, including alterations in insulin signaling [2]. One of the most important actions of insulin that is specific to the endothelium is the nitric oxide (NO) synthesis, dependent on the IR/IRS1/PI3K/Akt pathways and the activation by phosphorylation of eNOS at Ser 1177 [3,4]. Insulin-induced NO production enhances smooth muscle vasodilation and blood flow, which are impaired in the insulin-resistant state [5]. Given the vasotropic functions of insulin in endothelial cells, endothelial insulin resistance results in an impaired endothelial-dependent vasodilation, which represents a common feature of endothelial dysfunction in diabetic patients, and an important cause of complications, responsible for the high mortality associated to T2DM [6]. Therefore, there is a critical need to better understand the underlying mechanisms responsible for the endothelial insulin resistance, in order to develop new and improved therapeutic strategies for these chronic conditions.

Besides genetics, environmental and epigenetic modifications act as major contributors to the onset of T2DM and its associated complications. Recent studies have indeed demonstrated clear links between altered miRNA expression and vascular dysfunction, implicating certain miRNAs in the development of diabetes-related injury in the heart, kidney, peripheral nerves, and retina [6]. MicroRNAs (miRNAs) are a class of noncoding, single-stranded RNA molecules containing 20–22 nucleotides. They are ubiquitously expressed, and regulate several crucial biological pathways and cellular functions by affecting post-transcriptional gene expression [7]. miRNAs have emerged as key epigenetic regulators of metabolism, and their deregulation contributes to metabolic diseases, including T2DM [8]. Zampetaki et al. [9] profiled miRNAs in the plasma of 800 patients in a prospective study of T2DM patients, and established a diabetes signature based on 10 miRNAs. Interestingly, the reduced expression of 6 of these miRNAs several years before the onset of T2DM, correlated with subclinical peripheral artery disease, suggesting their use as markers in patients at risk for diabetic complications. miRNAs stand out also as a contributing factor in the development of insulin resistance, which plays an important role in glucose homeostasis and endothelial function. miRNAs have been described to influence insulin signal transduction by translation inhibition of insulin-signaling mediators [10,11,12,13], or by regulation of their modulators, such as caveolin 1, which is essential for insulin receptor regulation [14], or the phosphatase PHLPP2 that de-phosphorylates Akt at Ser473 [15].

Methylglyoxal (MGO) is a major precursor of advanced glycated end-products (AGEs), which represent the most important mechanism of metabolic memory that underlies the pathophysiology of chronic diabetic complications [16]. MGO is mainly generated as side-product of glycolysis, by the degradation of a smallest amount (1%) of triose phosphates. MGO levels are increased up to 5-fold in diabetic patients. Independently of the level of hyperglycemia, MGO mediates intracellular glycation of proteins, and is associated with vascular dysfunction [17]. Recently, several miRNAs have been identified that interfere with and modulate intracellular AGE signaling in the context of diabetic micro- and macrovascular complications [16]. Moreover, MGO is also emerging as a modulator of gene expression through miRNA regulation [18,19].

We have previously demonstrated that high MGO levels lead to endothelial insulin resistance with impaired Akt-dependent pathway and NO release in response to insulin [20]. This effect is at least partly mediated by the miR-190a downregulation-dependent increase of Kirsten Rat Sarcoma Viral Proto-Oncogene (KRAS) levels in mouse aortic endothelial cells (MAECs). By our previous investigations, we have also found that the expression of other three miRNAs is related to diabetes: miR-214, miR-450a, and miR-126 are reduced by 32%, 22%, and 30%, respectively, in MAECs exposed to MGO [21]. The aim of this study is to analyze the contribution of these miRNAs in the effect induced by MGO on the insulin responsiveness in MAECs. 

## 2. Results

**Figure 1:**
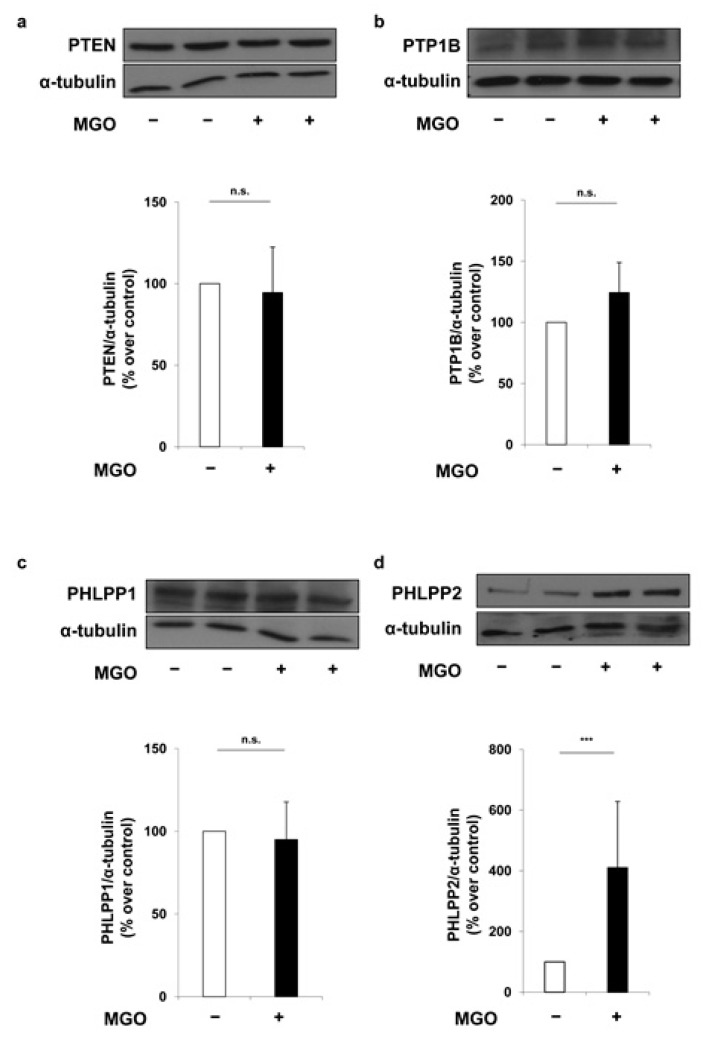
Effect of methylglyoxal (MGO) on miR-214 and miR-126 target phosphatases of insulin signaling mediators. Mouse aortic endothelial cells (MAECs) were treated with 500 μmol/L MGO for 16 h. Protein lysates obtained from these cells were analyzed by Western blot with anti-PTEN (**a**) anti-PTP1B (**b**) anti-PHLPP1 (**c**) and anti-PHLPP2 antibodies (**d**). Protein normalization was performed using α-tubulin antibody. Exposure timing was of 1 min for blots in panels (**a**,**c**,**d**), and 2 min for blots in panel (**b**). Protein levels were quantified by the densitometric analysis of at least three independent experiments. Bars in the graphs represent the mean ± SD of the percent (%) over control (−MGO). Statistical analysis was evaluated using the Student’s *t*-test; *** *p* ≤ 0.001.

In order to evaluate the possible contribution of miRNAs to insulin signal transduction, found to be altered in MAECs exposed to MGO [18], a combination of available algorithms has been considered, and a comprehensive atlas of the putative miRNA binding site predictions has been consulted using the mirWalk database. This search has allowed the identification of candidate genes involved in insulin signal transduction as potential targets of miR-214 and miR-126. Among these, the phosphatase and tensin homolog (PTEN), protein tyrosine phosphatase 1B (PTP1B), PH domain leucine-rich repeat protein phosphatase 1 and 2 (PHLPP1 and PHLPP2) play an important role in the downregulation of insulin signaling. As shown in Figure 1, treatment of MAECs with 500 μmol/L MGO has no effect on the protein levels of PTEN (Figure 1a), PTP1B (Figure 1b) and PHLPP1 (Figure 1c). Conversely, protein levels of PHLPP2 are 4-fold increased in MGO-treated MAECs compared to the untreated control cells (Figure 1d).

**Figure 2:**
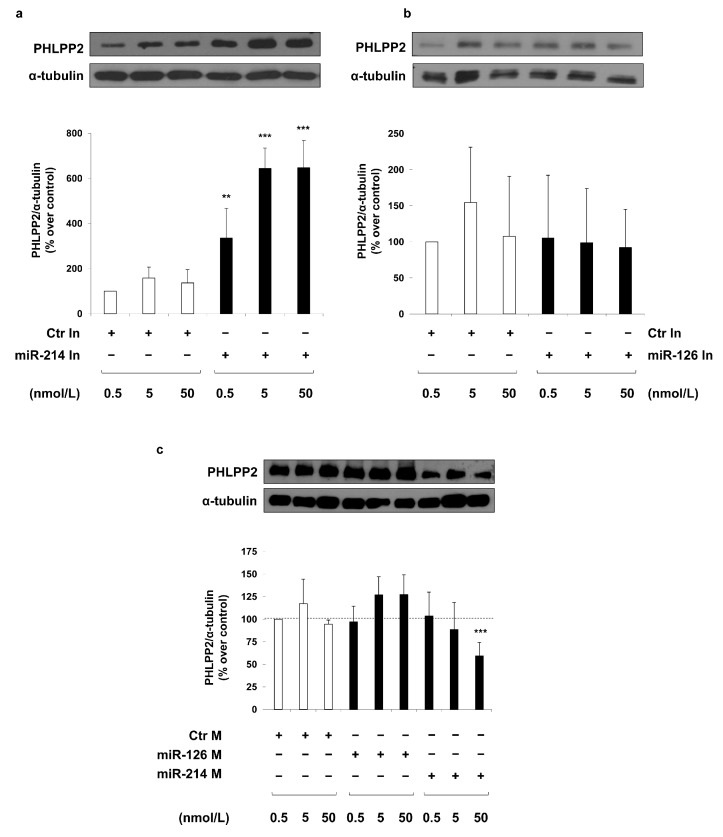
Effect of miR-214 and miR-126 modulation on PHLPP2 protein levels in MAECs. MAECs were transfected with different concentrations (0.5, 5, and 50 nmol/L) of miR-214 (**a**) and miR-126 (**b**) inhibitor (miR-214 In and miR-126 In), or miR-214 and miR-126 mimic (miR-214 M and miR-126 M) (**c**). Transfection with a non-targeting antisense inhibitor (Ctr In; (**a**,**b**)), and a non-targeting control oligonucleotide (Ctr M; (**c**)) were used as negative controls. Forty-eight hours after transfection, cells were collected and protein lysates were analyzed by Western blot with anti-PHLPP2 and anti-α-tubulin antibodies. Exposure timing was 4 min for blots in panels (**a**), 2 min for blots in panels (**b**) and 20 min for blots in panel (**c**). Protein levels were quantified by the densitometric analysis of at least three independent experiments. Bars in the graphs represent the mean ± SD of the percent (%) over control (Ctr In or Ctr M) 0.5 nmol/L. Statistical analysis was evaluated using Student’s *t*-test; ** *p* ≤ 0.01; *** *p* ≤ 0.001.

Interestingly, transfection of a specific antisense inhibitor for miR-214 (miR-214 inhibitor) induces a dose-dependent increase of PHLPP2 protein levels in MAECs (Figure 2a). Whereas, no difference is observed between MAECs transfected with a specific antisense inhibitor for miR-126 (miR-126 inhibitor) and MAECs transfected with a non-targeting antisense inhibitor (control inhibitor) (Figure 2b). In accordance with the previous results, a significant reduction in PHLPP2 levels is observed in MAECs overexpressing miR-214 with the transfection of the highest concentration (50 nmol/L) of a RNA oligonucleotide which specifically mimics miR-214 (miR-214 mimic). By contrast, no effect is obtained in MAECs transfected with a RNA oligonucleotide specific for miR-126 in comparison with cells transfected with a non-targeting control oligonucleotide (control mimic) (Figure 2c). Taken together, these results show that miR-214, but not miR-126, is able to modulate the protein levels of PHLPP2. 

**Figure 3:**
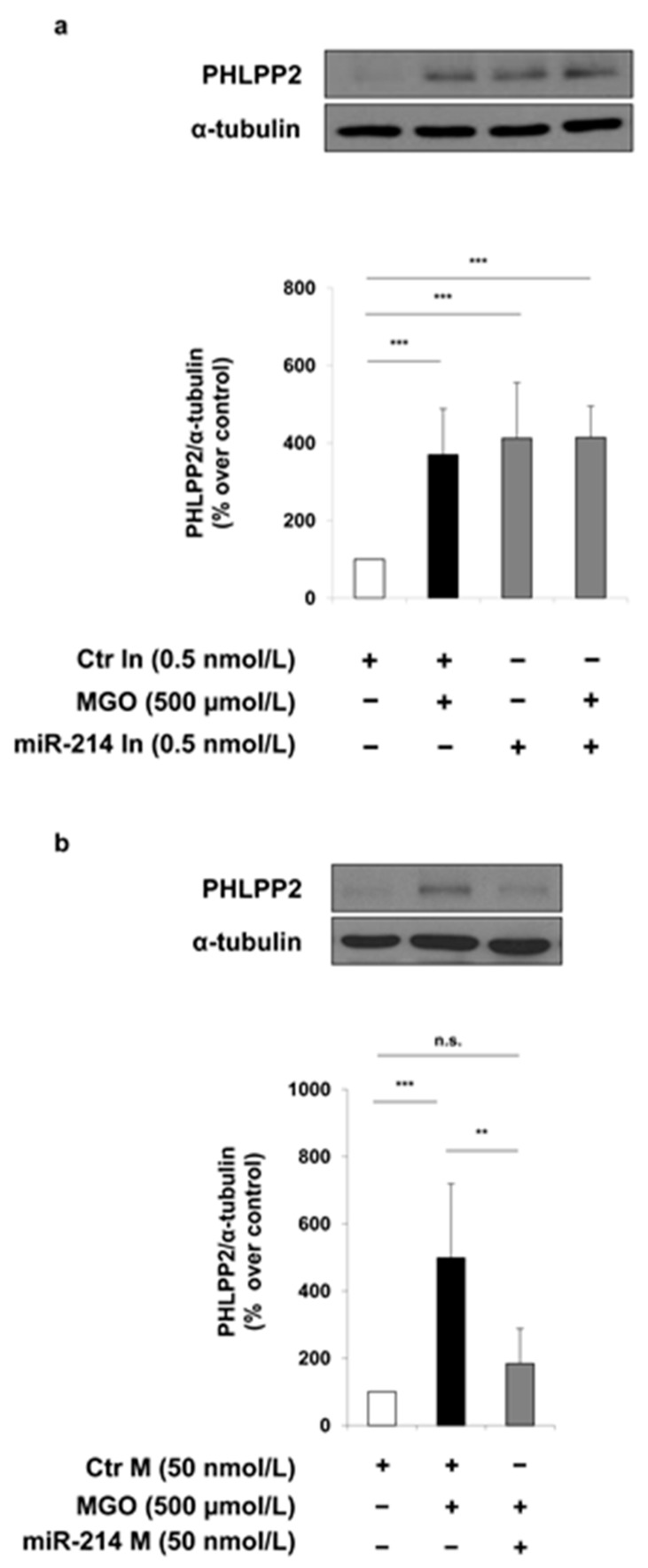
Effect of miR-214 mimic and miR-214 inhibitor on PHLPP2 levels in MAECs with or without MGO. MAECs were transfected with a non-targeting antisense inhibitor (Ctr In 0.5 nmol/L; (**a**)) and a non-targeting control oligonucleotide (Ctr M 50 nmol/L; (**b**)) (white and black bars), with miR-214 inhibitor (miR-214 In 0.5 nmol/L; (**a**)) or miR-214 mimic (miR-214 M 50 nmol/L; (**b**)) (gray bars). Where indicated, MAECs were treated with 500 μmol/L MGO for 16 h. Protein lysates obtained from these cells 48 h after transfection were analyzed by Western blot with anti-PHLPP2 and anti-α-tubulin antibodies. Exposure timing was of 2 min for PHLPP2 blots and 10 min for α-tubulin blots. Protein levels were quantified by the densitometric analysis of at least three independent experiments. Bars in the graphs represent the mean ± SD of the percent (%) over control (Ctr M and Ctr In). Statistical analysis was evaluated using the Student’s *t*-test; ** *p* ≤ 0.01; *** *p* ≤ 0.001.

In MAECs transfected with the miR-214 inhibitor, PHLPP2 levels are increased by about 4-fold, and are comparable both to that observed in MGO-treated control cells and miR-214 inhibitor plus MGO co-treated cells (Figure 3a). In addition, in the presence of MGO, the transfection of MAECs with the miR-214 mimic reverses the effect due to MGO, leading to a 2.5-fold reduction of PHLPP2 (Figure 3b). Therefore, these results demonstrate that miR-214 mediates the effect of MGO on the PHLPP2 expression. 

**Figure 4:**
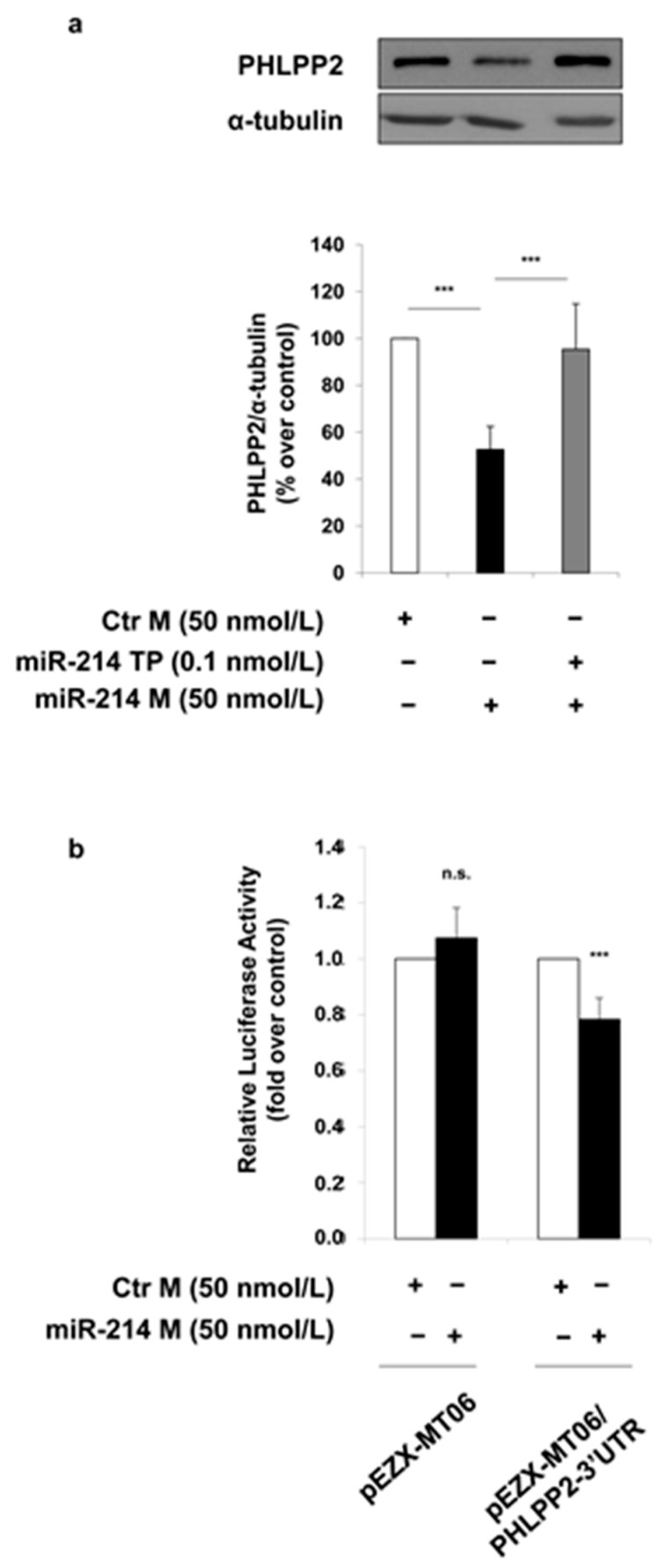
Effect of miR-214 on PHLPP2 regulation. (**a**) MAECs were transfected with a non-targeting control oligonucleotide (Ctr M; white bars) or miR-214 mimic (miR-214 M 50 nmol/L; black bars). Where indicated, MAECs were co-transfected with miScript miR-214 Target Protector (miR-214 TP 0.1 nmol/L). Protein lysates obtained from 48 h transfected MAECs were analyzed by Western blot with anti-PHLPP2 and anti-α-tubulin antibodies. Exposure timing of blots was of 2 min. Protein levels were quantified by the densitometric analysis of at least three independent experiments. Bars in the graphs represent the mean ± SD of the percent (%) over control (Ctr M). (**b**) MAECs were transfected for 24 h with non-targeting control oligonucleotide (Ctr M 50 nmol/L; white bars) or miR-214 mimic (miR-214 M 50 nmol/L; black bars) in the presence of the pEZX-MT06 control reporter vector or the pEZX-MT06/PHLPP2-3′UTR reporter vector. Firefly and Renilla luciferase activities were determined in cell lysates under each experimental condition. Results are normalized to Renilla activity. Bars in the graphs represent the mean ± SD of the fold over control (Ctr M). Statistical analysis was evaluated using Student’s *t*-test; *** *p* ≤ 0.001.

In order to prove a direct regulation of PHLPP2 expression by miR-214, a miScript Target Protector (TP) specific for the binding site of miR-214 on the 3′ untranslated region (UTR) of the mouse PHLPP2 has been used. The decrease in the protein levels of PHLPP2, which is induced by miR-214 mimic, is no longer observed in MAECs transfected with both the miScript Target Protector (TP) and miR-214 mimic (Figure 4a). Furthermore, a miR-214-dependent regulation of PHLPP2 was confirmed by luciferase reporter assays, performed in MAECs using a construct in which the PHLPP2-3′UTR is cloned immediately downstream of the Firefly luciferase. The transfection of miR-214 mimic induces a 25% reduction of luciferase activity driven by the PHLPP2-3′UTR, while no effect on luciferase activity is induced by miR-214 mimic in the empty control vector-transfected cells (Figure 4b), demonstrating that miR-214 binds the PHLPP2-3′UTR region.

**Figure 5:**
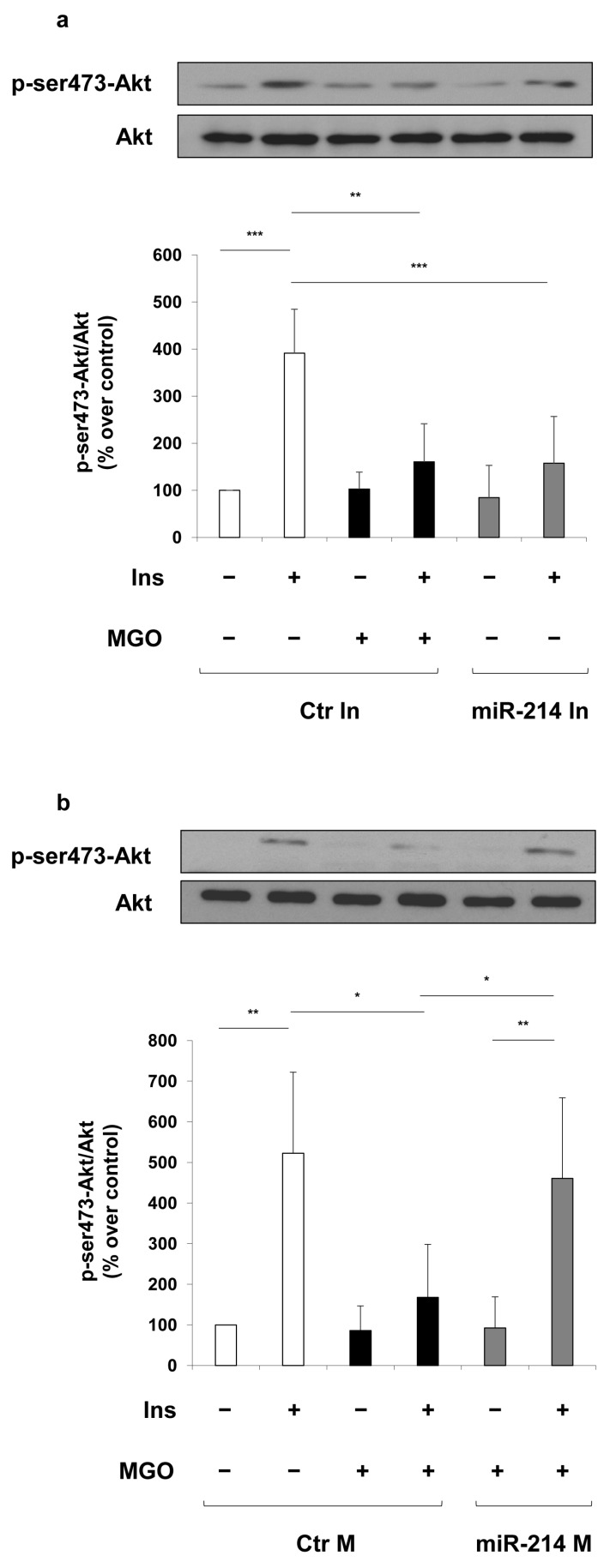
Effect of miR-214 modulation on insulin-dependent Akt activation in MAECs. MAECs were transfected with a non-targeting antisense inhibitor (Ctr In 0.5 nmol/L; (**a**)) or a non-targeting control oligonucleotide (Ctr M 50 nmol/L; (**b**)) (white and black bars), with miR-214 inhibitor (**a**) or miR-214 mimic (**b**) (miR-214 In 0.5 nmol/L or miR-214 M 50 nmol/L; gray bars). Where indicated, MAECs were treated with 500 μmol/L MGO (black bars) for 16 h before harvesting. After 48 h of transfection, cells were stimulated or not with 100 nmol/L insulin for 10 min. Protein lysates were analyzed by Western blot with p-ser473-Akt and Akt antibodies. Exposure timing was of 30 seconds. Protein levels were quantified by the densitometric analysis of at least three independent experiments. p-ser473-Akt levels were normalized to Akt total levels. Bars in the graphs represent the mean ± SD of the percent (%) over control (Ctr In and Ctr M). Statistical analysis was evaluated using the Student’s *t*-test; * *p* ≤ 0.05; ** *p* ≤ 0.01; *** *p* ≤ 0.001.

Finally, to prove the role of miR-214 on endothelial MGO-mediated insulin-resistance, we analyzed the effect of miR-214 inhibitor and miR-214 mimic on insulin-dependent phosphorylation of Akt. As shown in Figure 5, MGO treatment in MAECs transfected with control inhibitor induces a reduction of insulin-induced Akt phosphorylation compared to the same cells not exposed to MGO. Similarly, the transfection of MAECs with miR-214 inhibitor significantly decreases insulin-stimulated Akt phosphorylation compared to MAECs transfected with control inhibitor (Figure 5a). Differently, the transfection of miR-214 mimic prevents the MGO deleterious effect on the insulin-dependent Akt phosphorylation (Figure 5b).

**Figure 6:**
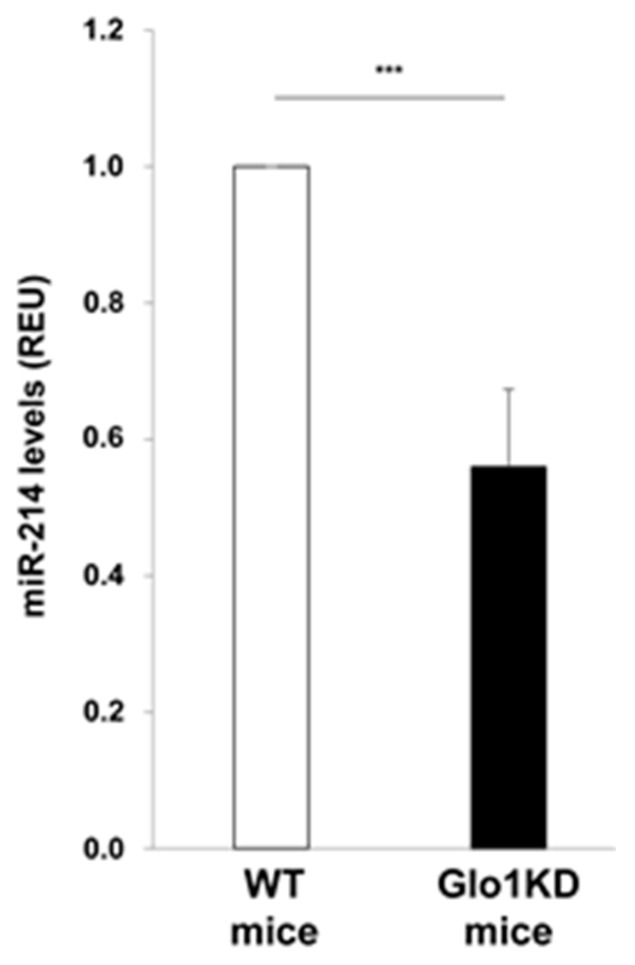
Effect of MGO on miR-214 levels in mouse aortic tissue. miR-214 levels were evaluated by real time-PCR in aortae isolated from Glo1KD (*n* = 5) and WT (*n* = 8) mice. Bars in the graph represent the mean ± SD of the expression units relative to U6 snRNA levels, used as housekeeping small RNA. Statistical analysis was evaluated using the Student’s *t*-test; *** *p* ≤ 0.001.

Finally, miR-214 downregulation was also validated in vivo in the aortic tissue of a non-diabetic mouse model knocked down for glyoxalase 1 (Glo1KD mice), described to have high levels of MGO-modified proteins [22,23]. Figure 6 shows that miR-214 expression is reduced by 45% in the aortic tissue of Glo1KD mice compared to wild type (WT) mice. 

## 3. Discussion

Accumulating evidence suggests that endothelial dysfunction is a direct consequence of, and a common feature associated with T2DM. In order to better understand how vascular damage develops, particularly in T2DM individuals, studies on the function of the endothelium in health and disease have been conducted, and several mechanisms pertaining to inflammation and insulin resistance have been proposed [24,25,26]. Indeed, elucidating the mechanisms responsible for the diabetes-associated endothelial dysfunction is highly important, and will have a substantial clinical impact on future translational and interventional research.

Recently, several miRNAs have emerged as key regulators of endothelial function and their alteration has been demonstrated in diabetes-induced endothelial dysfunction, contributing to inflammation, insulin resistance, hyperglycemia, angiogenesis and endothelial senescence, which are the principal mechanisms in endothelial dysfunction [27,28]. Hence, normalizing the expression of the dysregulated miRNAs may serve as a therapeutic approach in controlling the development and/or progression of endothelial dysfunction.

Methylglyoxal (MGO) accumulates in the cells under diabetic conditions and contributes to insulin resistance [17,20]. We and others have recently demonstrated that MGO affects miRNA expression in endothelial cells [21,29]. Indeed, in our previous work, we performed a miRNA array, by which we evaluated the expression profile of 84 miRNAs related to diabetes, already reported in literature. We confirmed that 4 out of these 84 miRNAs were significantly altered in MGO-treated MAECs: miR-126, miR-190a, miR-214 and miR-450a [21]. 

The working hypothesis is that these miRNAs altered by MGO may activate alternative mechanisms, thus contributing, in different ways, to the impairment of the endothelial insulin sensitivity due to MGO. We have already demonstrated that miR-190a negatively affects the endothelial insulin sensitivity through KRAS [21]. For the present study, both miR-214 and miR-126 appear to be attractive candidates, since they control endothelial cell function and angiogenesis [30,31,32,33,34,35]. While very little is known about miR-450a to support its selection as a candidate for further investigation on its potential role in MGO-treated MAECs, it has been shown that miR-126 is able to downregulate IRS-1, suppressing Akt activation [36,37]. Moreover, miR-214 has been reported to activate the PI3K/Akt signaling by targeting PTEN expression in several cell types, including ovarian and gastric cancer cells [38,39,40,41,42,43]. It has been also shown that miR-214 targets IRS and Akt in C2C12 myoblasts [44]. 

Regarding the search for new targets of the altered miRNAs in MGO-treated MAECs, in light of our previous investigations, we have excluded key components of the insulin signaling pathway. Indeed, we previously examined the protein levels of IRS-1, Akt, and eNOS, which are potential targets of the aforementioned miRNAs, but no changes have been observed for these mediators [20,21]. Therefore, we have shifted our focus towards negative regulators of the insulin signaling, since their upregulation due to the downregulated miRNAs by MGO would provide a reasonable explanation for the decrease of insulin responsiveness observed in MGO-treated MAECs. 

Employing an integrated approach using public bioinformatics tools, we have identified several phosphatases as potential targets of both miR-214 and miR-126. For this reason, we have tested the effects of MGO on the levels of the following proteins: PTP1B, which binds and subsequently dephosphorylates the insulin receptor [45]; the lipid phosphatase PTEN, which dephosphorylates PIP3 and antagonizes the PI3K signaling [46]; and the serine phosphatases PHLPP1 and PHLPP2, which specifically dephosphorylate Akt at the serine 473 residue and inactivate it [47,48,49]. Upon treatment with MGO, we have observed no alterations in the protein levels of PTP1B and PHLPP1 in MAECs. Surprisingly, despite PTEN being a widely validated target of miR-214 [38,39,40,41,42,43], its levels have not been altered by MGO in MAECs. One possible explanation for the lack of PTEN regulation by miR-214 in our cellular model is that the binding site for miR-214 in the 3′UTR of mouse PTEN overlaps with the binding site for several other miRNAs in accordance with the microRNA.org prediction, suggesting that other miRNAs highly expressed in MAECs may regulate PTEN in a mutually exclusive fashion and independently from any changes in miR-214 levels upon MGO treatment. 

In our search, PHLPP2 is increased by MGO treatment in MAECs. PHLPP2 is a high confidence predicted target of both miR-214 and miR-126, based on our search using 10 different miRNA target prediction programs to retrieve information on any interaction between PHLPP2 and these specific miRNAs. We obtained positive information from 8 and 7 programs for miR-214 and miR-126, respectively. Interestingly, the inhibition of the endogenous miR-214 in MAECs is sufficient to cause a significant increase in PHLPP2 protein levels, comparable to that obtained upon MGO treatment; in addition, when miR-214 is overexpressed, the phosphatase levels are significantly decreased. On the contrary, by modulating the miR-126 levels in MAECs, no alterations in the PHLPP2 protein levels are observed. Taken together, these results suggest an inverse correlation between PHLPP2 and miR-214, but not with miR-126, supporting the bioinformatic target prediction only for miR-214. Furthermore, for the first time, the direct regulatory effect of miR-214 on the PHLPP2 expression has been verified using both a target site protector specific for the binding site of miR-214 in the 3′UTR of mouse PHLPP2 and a reporter gene assay with the 3′UTR of mouse PHLPP2 cloned downstream of the Firefly luciferase gene. 

In order to strengthen the role of miR-214 in the MGO effect on the PHLPP2 levels in MAECs, this miRNA has been both inhibited and overexpressed in presence of MGO. While no further increase is observed in the former case, thus excluding that two different mechanisms are due to miR-214 and MGO, a reversion in the PHLPP2 expression is obtained when the MGO-dependent downregulation of miR-214 is specifically bypassed in the latter case.

Considering PHLPP2 as a direct target of miR-214, we subsequently verified whether the negative effect of MGO on the insulin signaling pathway in MAECs may be mediated by miR-214 downregulation. To this end, we evaluated the Akt phosphorylation levels on serine 473 following the specific inhibition of miR-214 in MAECs. As expected from the increased PHLPP2 levels, a decrease in the phosphorylation levels of Akt is observed in these conditions, thus mimicking the MGO-dependent effect on the insulin sensitivity in the endothelial cells. In addition, bypassing the MGO-dependent downregulation of miR-214 by miR-214 mimics transfection in MAECs, a rescue effect of Akt phosphorylation as well as PHLPP2 levels is observed, strengthening our hypothesis that the downregulation of miR-214 may contribute to the MGO-dependent effect through its new target, PHLPP2. Furthermore, we have provided the first evidence that high MGO levels are associated with 45% of the miR-214 reduction in aortic tissues from Glo1KD mice, in accordance with an impaired insulin sensitivity previously observed in these mice when compared to their WT littermates [21]. Indeed, this mouse model is suitable for the in vivo study of the MGO-dependent effects, as its characteristic feature is represented by reduced levels of Glo1 that are responsible for the accumulation of MGO-modified proteins and the development of diabetic complications in the absence of hyperglycemia [22,23].

Studies have already shown that PHLPP2 can directly dephosphorylate Akt to inhibit its signaling activity in endothelial cells [14]. In this study, we have demonstrated that miR-214 negatively affects the responsiveness to insulin in MAECs by interacting with PHLPP2, which is a novel finding not previously reported to the best of our knowledge.

## 4. Materials and Methods 

### 4.1. Reagents

Media, sera and antibiotics for cell culture were from Lonza (Walkersville, MD, USA). MGO (40% in water) was from Sigma-Aldrich (St Louis, MO, USA). Insulin was from Eli Lilly (Florence, Italy). The antibodies used were anti-p-ser473-Akt and anti-PTEN (Cell Signaling Technology, Beverly, MA, USA), anti-α-tubulin (Sigma-Aldrich), anti-PHLPP1 and anti-PHLPP2 (Bethly Laboratories, Montgomery, TE, USA), anti-PTP1B and anti-Akt (Santa Cruz, CA, USA). All other chemicals were from Sigma-Aldrich (St Louis, MO, USA). Protein electrophoresis and Western blot reagents were from Bio-Rad (Richmond, VA, USA) and chemiluminescence reagents from Pierce (Rockford, IL, USA). 

### 4.2. Cell Culture

Mouse aortic endothelial cells (MAECs) were kindly provided by Thomas Henry Fleming (University of Heidelberg, Heidelberg, Germany) and cultured as previously described [20]. Briefly, MAECs were plated in T75 flasks and grown in Dulbecco’s Modified Medium (DMEM) containing 1 g/L glucose supplemented with 10% (*v*/*v*) FBS, 2 mmol/L l-glutamine and 0.1 mmol/L non-essential amino acids. Cell cultures were maintained at 37 °C in a humidified atmosphere containing 5% (*v*/*v*) CO_2_. MGO exposure was performed for 16 h in MAECs by adding MGO to culture medium at the final concentration of 500 μmol/L. Where indicated, MAECs were stimulated with 100 nmol/L insulin for 10 min, following starvation in serum-free medium containing 0.25% (*w*/*v*) albumin bovine serum (BSA) for 16 h. 

### 4.3. miRNA Target Prediction

miRWalk2.0, a comprehensive atlas of microRNA–target interactions, was used [50] for target prediction of specific miRNAs. 

### 4.4. miRNA Mimic and Inhibitor Transfection

miR-214 levels were modulated by transfecting MAECs with 0.5, 5, or 50 nmol/L miR-214-mimic (mmu-miR-214 miRIDIAN Mimic) or miR-214-inhibitor (mmu-miR-214 miRIDIAN Harpin Inhibitor). miR-126 levels were modulated by transfecting MAECs with 0.5, 5 and 50 nmol/L miR-126-mimic (mmu-miR-126 miRIDIAN Mimic) or miR-126-inhibitor (mmu-miR-126 miRIDIAN Harpin Inhibitor). miRIDIAN microRNA Mimic negative control #1 and miRIDIAN microRNA Harpin Inhibitor negative control #1 (Dharmacon, Lafayette, CO, USA) were used as negative controls of miRNA-mimic and miRNA-inhibitor transfection, respectively. DharmaFECT 4 was used as transfecting agent (Dharmacon), according to the manufacturer’s instructions. Forty-eight hours after transfection, cells were harvested and proceeded as described below. 

### 4.5. Western Blot Analysis

MAECs were solubilized in lysis buffer (50 mmol/L HEPES pH 7.5, 150 mmol/L NaCl, 10 mmol/L EDTA, 10 mmol/L Na_2_P_2_O_7_, 2 mmol/L Na_3_VO_4_, 100mmol/L NaF, 10% glycerol, 1% Triton X-100) for 2 h at 4 °C. Protein lysates were clarified by centrifugation at 16,000× g for 20 min. Cell lysates were then separated by SDS-PAGE and transferred into 0.45 μm Protran Nitrocellulose Membrane (Sigma-Aldrich). Upon incubation with primary and secondary antibodies (for a full list of the antibodies, see above), immunoreactive bands were detected by chemiluminescence and densitometric analysis was performed using ImageJ software.

### 4.6. miRNA Reverse Transcription, miScript PCR Array, and Real Time-PCR

Total RNA was isolated from the aortic tissue of Glo1-knockdown (Glo1KD) mice [21], after homogenization in Qiazol using miRNeasy mini kit (QIAGEN, Hilden, Germany), according to manufacturer’s instructions. After quantification with NanoDrop 2000 spectrophotometer (Thermo Scientific, Waltham, MA, USA), total RNA was reverse transcribed using the miScript II RT Kit (QIAGEN), and the differential expression of miRNA-214 was analyzed by real time-PCR using the miScript SYBR Green PCR Kit (QIAGEN) and quantified as expression units relative to U6 snRNA, used as housekeeping small RNA. 

Specific primers used for amplification were purchased from QIAGEN:

Mm_miR-214_2 miScript Primer Assay, MS00032571
RNU6B_13 miScript Primer Assay, MS00014000

### 4.7. Target Site Inhibition Assays

The miScript miR-214 Target Protector (miR-214 TP) was designed based on the sequence of the target site for the miR-214 in the 3′UTR of the mouse PHLPP2 mRNA and obtained from Qiagen (Qiagen, Germantown, MD, USA). The miScript Target Protector is a single-stranded modified RNA that is complementary to the binding site of the miRNA of interest and covers the flanking region of the binding site, thus interfering specifically with the interaction of the miRNA with a single target and leaving the regulation of other targets of the same miRNA unaffected. After transfection, a miScript Target Protector binds to its specific miRNA-binding site, blocking miRNA access to the site and preventing gene downregulation by a specific miRNA.

MAECs were co-transfected with both 0.1 nmol/L miR-214 TP and 50 nmol/L miR-214 mimic using DharmaFECT 4 (Dharmacon), according to manufacturer’s instructions. Forty-eight hours after transfection, cells were harvested and PHLPP2 protein levels were analyzed as described below.

### 4.8. Luciferase Reporter Assays

The mouse PHLPP2-3′UTR sequence was inserted into pEZX-MT06, a Firefly/Renilla Duo-Luciferase reporter vector (GeneCopoeia, Rockville, MD, USA) [51]. Here, Firefly luciferase is the reporter gene controlled by the 3′UTR of interest and Renilla luciferase is the internal control.

MAECs were plated in 6-well plates and co-transfected with 100 ng of the pEZX-MT06 control reporter vector or the pEZX-MT06/PHLPP2-3′UTR reporter vector and 50 nmol/L miR-214 mimic or a non-targeting control oligonucleotide (mimic control) using DharmaFECT 4 (Dharmacon), according to manufacturer’s instructions. Twenty-four hours post transfection, Firefly and Renilla luciferase activities were determined in cell lysates using the Dual-Luciferase Reporter Assay System (Promega, Madison, WI, USA) and a luminometer (Orion I, Berthold Detection Systems, Pforzheim, Germany) according to the manufacturer. Results were expressed as the ratio of Firefly to Renilla activity. The experiments were performed in triplicate.

### 4.9. Statistic Procedures

Data are expressed as means ± SD, as indicated in figure legends. Comparison between groups were performed using Student’s *t*-test. A *p*-value of less than 0.05 was considered statistically significant.

## 5. Conclusions

In conclusion, for the first time, this study provides evidence that an essential link exists between miR-214 and insulin resistance and between miR-214-dependent insulin resistance and PHLPP2 in endothelial cells. Hence, these findings highlight an important role for miR-214 under diabetic conditions and suggest that strategies aimed at restoring miR-214 levels or inhibiting PHLPP2 protein levels in endothelial cells may provide the basis for the rationale design of novel therapies for insulin resistance and vascular complications.

## Abbreviations

MGO	methylglyoxal
miRNAs	microRNAs
NO	nitric oxide
PTEN	phosphatase and tensin homolog
PTP1B	protein tyrosine phosphatase 1B
PHLPP1	PH domain leucine-rich repeat protein phosphatase 1
PHLPP2	PH domain leucine-rich repeat protein phosphatase 2
TP	target protector
T2DM	type 2 diabetes mellitus
UTR	untranslated region
